# On English proverb variation from the perspective of linguistic creativity

**DOI:** 10.3389/fpsyg.2023.1213649

**Published:** 2023-07-05

**Authors:** Jianhao Wu, Wanting Zhou, Bin Shao

**Affiliations:** ^1^Culture and Art School, Zhejiang Technical Institute of Economics, Hangzhou, China; ^2^School of Foreign Languages, Zhejiang University of Finance and Economics, Hangzhou, China; ^3^School of International Studies, Zhejiang University, Hangzhou, China

**Keywords:** English proverb, proverb variation, linguistic creativity, cognitive creativity, EFL learner

## Abstract

Proverbs are usually regarded as structurally fixed expressions. However, in daily communication, language users often change them to suit their communicative purposes in many ways, resulting in proverb variations. Using the data from the Corpus of Contemporary American English (COCA corpus), this study attempts to present varieties of the English proverb “There are two sides to every coin” and explain the variations from the perspective of linguistic creativity. This study also explores the variations of this proverb in EFL learners' use via the data from Chinese EFL learners' corpus TECCL. The study shows that, first proverb use can roughly be divided into two types: canonical and non-canonical uses, each having three ways of alteration, i.e., addition of modifiers, substitution of content words, and reduction. Second, Chinese EFL learners tend to use the proverb in a mechanical way with little variation, which shows their inflexible use of proverbs. Finally, proverb variation by nature is the creative manipulation of language use to fit the context, which is a form of linguistic creativity that reflects the cognitive creativity of human beings.

## Introduction

Proverbs are familiar, fixed, sentential expressions that express well-known truths, social norms, or moral concerns (Gibbs and Beitel, 1995). By definition, proverbs are fixed in structure. In practice, however, proverbs are open-ended and language users can make many changes to proverbs in accordance with their communication needs (Fernando, 2000, p. 43). This is known as proverb variation. As pointed out by Steyer (2014, p. 214), one of the key questions in proverb studies and proverb lexicography is about the fixedness and variance of proverbs. The lexical and grammatical variation in proverbs has been explored in previous studies (Norrick, 1981, 1985; Mieder, 2004). Some scholars also investigated proverb usage by non-native speakers. For example, Cignoni et al. (1999) compared the variations of the same proverb in the native English corpus and the Italian English learners' corpus. However, there are some limitations in the previous studies. One is that little research has been carried out on Chinese EFL learners' use of English proverbs. The other is that few studies have examined proverb variations from the perspective of linguistic creativity. Given these research gaps, this study attempts to examine the usage of the proverb *There are two sides to every coin* based on the Corpus of Contemporary American English (henceforth COCA) and Ten-thousand English Compositions of Chinese Learners Corpus (henceforth TECCL) to summarize its variations as used by native speakers and Chinese EFL learners, and explore the proverb variations from the perspective of linguistic creativity.

### Variation of idioms and proverbs

A proverb is a “proverbial idiom,” which is opposite to the “lexemic idiom” (Makaai, 1972). The latter is a phrase, such as *kick the bucket*, while the former is a sentence, such as *Don't count your chicken before they are hatched*. As a type of idiomatic expression, English proverbs share certain basic characteristics with idioms. Moon (1998, p. 6) puts forward her criteria of fixed expressions and idioms (FEIs), including proverbs in English, i.e., the process of ‘institutionalization', the word group's lexicogrammatical fixedness (also called “formal rigidity”), and their non-compositionality (to some degree).

As for the institutionalization of FEIs, McMordie (1954) holds the view that one cannot make any changes to English FEIs like substituting a word with its synonym or changing the word order. He argues that any minor change or modification to idioms may result in a loss of their inherent significance and ultimately cancel their communicative power.

The so-called “rigidity of form,” according to Taylor (1962, p. 135), is an essential characteristic of proverbs. Norrick (1985, p. 43, 70) views the fixed form as one defining property of the proverb, in that it distinguishes proverbs from free phrase combinations; he also agrees to label proverbs as “fixed expressions.”

Non-compositionality means the meaning of a FEI cannot be derived directly from the meaning of its components. Proverbs, as epigrammatic sentences expressing popular wisdom, a truth, or a moral lesson, in most cases contain meanings that are quite different from or much deeper than the sum of literal meanings of their components.

However, Moon (1998, p. 123) finds that FEIs, proverbs included, show a range of variations. In the following part, we will review the literature on the variations of idioms and proverbs.

In practice, the forms of an idiom may vary based on different contexts (Cowie and Mackin, 1975). For example, Philip (2006, p. 95–108) examines idiomatic variants based on corpora and proposes the concept of “phraseological skeleton,” referring to the core part of idiomatic variants which remains unchanged. Duffley (2013) collects the data of two idioms, i.e., *kick the bucket* and *shoot the breeze*, and finds that they produce variation types through structural transformation and lexical variation. Some scholars have analyzed the mechanism behind the variation of idioms from grammatical and pragmatic perspectives. For example, O'Grady (1998) puts forward the continuity constraint principle, which indicates that the central word of an idiom hinders the added words within it from certifying the grammatical rationality of the variations. Vega-Moreno (2001) points out that the variation of idioms is a result of verbal changes in communication, and a variant needs to have the maximum correlation with the original idiom so that the listener can understand what it means.

Variation of proverbs is by no means a conceptional phenomenon. According to Fernando (2000, p. 43), most proverbs “can be manipulated or transformed in various ways according to the communicative needs of the language-user,” which means some parts of a proverb can be substituted, modified, inverted, or even deleted without changing its original implications. Steyer (2014, p. 214) argues that proverb variation occurs mainly on two levels: the syntactic and morphological level, and the lexical level. In addition, there is a special variant of proverbs—the anti-proverb, which means a distortion, parody, and misuse of the original proverb to create a comic or satirical effect (Doyle et al., 2012, p. 11). Proverbs and anti-proverbs are like two sides of the same coin, reflecting the interplay between conventional and innovative uses of language. Some scholars have explored proverb variation based on corpora. For instance, Moon (1998, p. 49) points out that proverbs often have variants and that the automatic search for variants of a proverb in a corpus is difficult and it often requires manual intervention. Rassi et al. (2014) uses natural language processing techniques to automatically search Brazilian Portuguese news texts, and the results show that proverb variants occur frequently. Other scholars have explored the variation of proverbs in different genres. For example, Konstantinova (2014, p. 282–283) explores the creative use of proverbs in mass media, dividing proverb variants into different types, such as temporary proverbs, anti-proverbs, and pseudo-proverbs. Doyle (2014, p. 264, 269–270) notes that in proverb poems (i.e., poems composed of proverbs), the proverbs were subject to necessary variations according to the pattern of a poem. Doyle also finds that proverb variants were often used as titles in literary texts. For example, the title *The Proof of the Pudding* comes from the proverb *the proof of the pudding is in the eating*. Although proverbs have different variations, the typology of proverb variation is by no means standardized in the literature.

In terms of the specific variation classifications, many studies focus on idiom variations. Cignoni et al. (1999) takes the idiom *rap someone on the Knuckle* as an example and summarizes the four types of variations: a basic form, lexical alternation (on/over), a change of focus (to rap vs. to get rapped), and a nominalization. Philip (2006, p. 221–233) conducts a corpus-based study of *like a red rag to a bull* and distinguishes the canonical form and variants. He finds that the variations of a proverb may include substitution, addition, or subtraction. In previous literature, there is no clear definition of “canonical use” and “non-canonical use” of the proverb. Philip (2006, p. 97) points out that the change from canonical form to variant, rather than being clear-cut, operates along a continuum. The “dictionary citation form” of a proverb is referred to as the canonical form. Lindquist (2009, p. 95) mentions “the so-called canonical complete form of the idiom.” Both authors emphasize completeness as the requirement for a canonical form. However, in the corpus, we find that native speakers do not always use proverbs in their full forms. Therefore, in the present study, the working definition for a canonical proverb is one that includes all the keywords of a proverb, such as “coin” and “sides” in our case. Other cases will be considered non-canonical.

### Linguistic creativity

Linguistic creativity has become a field of rapid development in linguistics. The creativity of language is, as Chomsky (1965, p. 6) puts it, “…one of the essential qualities that all languages have in common…. Thus, an essential property of language is that it provides the means for expressing indefinitely many thoughts and for reacting appropriately in an indefinite range of new situations.” However, following Lyons (1977), Bauer (1983, p. 63) makes a distinction between productivity and creativity. Productivity is that property of language that allows a native speaker to produce an infinitely large number of sentences, which is to be accounted for by the rules of generative grammar; creativity, on the other hand, is the language users' ability to extend the language system in a motivated, but unpredictable (non-rule-governed) way. In this sense, the so-called creativity in Chomsky's words is better to be named “productivity” (Yule, 2010, p. 13). Linguistic creativity in this study echoes Lyons (1977) and Bauer (1983), and it refers to the uses that are marked out as striking and innovative. More specifically, linguistic creativity involves a marked breaking or bending of rules and norms of language, including a deliberate play with its forms and its potential for meaning (Carter, 2004, p. 9).

The past two decades have witnessed a growing interest in everyday linguistic creativity. Carter (2004) points out that creativity is an all-pervasive feature of everyday language. Linguistic creativity reveals that speakers commonly make meanings in a variety of creative ways, in a wide range of social contexts, and for a diverse set of reasons. From a sociolinguistics perspective, Gillen (2018) investigates everyday linguistic creativity in communicative practices, deepening the understanding of everyday linguistic creativity as strategic performance in specific and complex contexts. Körtvélyessy et al. (2022) explore creativity in English word formation and word interpretation and link linguistic creativity with the general creative ability of human beings.

The proverb is part of everyday language, but few studies have explored its variation from the perspective of linguistic creativity. Furthermore, few scholars have analyzed the differences in their uses between English natives and EFL learners. Consequently, this study attempts to use the proverb *There are two sides to every coin* as a case study, categorize the types of proverb variants based on corpus data, and analyze them from the perspective of linguistic creativity. This proverb is one of the most frequently used proverbs by Chinese EFLs, as they prefer to use it to present their dialectical viewpoints in argumentative writing. Our research questions are as follows:

1) What are the differences in proverb use between native speakers and Chinese EFL learners?
2) What are the motivations behind proverb variations from the perspective of linguistic creativity?

## Data and methods

### Relevant corpora

This study makes a comparison between English natives' and EFL learners' use of proverbs and explores what is behind the difference. COCA is used to extract the data of native speakers. COCA contains more than one billion words of text, comprising over 25 million words each year from 1990 to 2019. The texts of COCA are divided into eight genres, namely spoken (SPOK), fiction (FIC), popular magazines (MAG), newspapers (NEWS), academic texts (ACAD), TV and Movies subtitles (TV/M), blogs (BLOG), and other web pages (WEB).

In order to see how English language learners and native speakers differ in their use of proverbs, this study applies TECCL to investigate the use of the proverb *There are two sides to every coin* and its variants by Chinese students. The TECCL corpus contains approximately 10,000 writing samples of Chinese EFL learners, totaling 1.8 million words. It should be mentioned that this study focuses on proverb variation used by the natives and learners, respectively, rather than on the overall frequency of the proverb, so the discrepancy in the size of the two corpora does not matter too much here.

### Data collection

According to *The Routledge Book of World Proverbs* (Stone, 2006), *There are two sides to every coin* is an American proverb, which means everything has two sides, positive and negative. The core words of the proverb are *coin* and *sides*, so variants of this proverb should meet the two criteria: containing the words *coin* or *sides* and relevant to the original proverb in meaning. Using the COCA online platform, we set “NOUN” in the lexical function of “Collocates” with the span [L4, R4] to obtain the nouns frequently collocated with *coin*. The result shows that the most frequent nouns are “side” and “sides.” Thus, the expressions that include “coin” and “side(s)” are potential variants of this proverb. After manually checking the data, a total of 1,210 items were obtained, of which 543 and 667 items contain the words *sides* and *side*. Moreover, considering that there are many nouns collocated with the word *sides* in COCA, we narrow the search scope to “there are two sides to/of ^*^ [n^*^]” to retrieve nouns that can replace *coin* and examine whether their meanings are closely related to the original proverb. Finally, we obtain a total of 137 items, such as *There are two sides to every story/argument*. Thus, the total number of proverbs in COCA is 1,347.

Among these 1,347 items, only 13 expressions contain the complete form of *There are two sides to every coin*, and the rest are more or less different from the original proverb. One is the canonical use, i.e., proverb variants that include the two core words *coin* and *sides*, and the other is non-canonical use, i.e., expressions containing only one core word, that is, a proverb with *coin* co-occurring with *side* or *sided*, or one that contains *sides* only. The canonical uses occur 482 times, accounting for 36% of the total, and the non-canonical uses occur 852 times, accounting for 64% of the total. Based on the specific variation methods, both the canonical and non-canonical categories can be divided into three subcategories in terms of their uses, namely addition of modifiers, substitution of content words, and reduction. Each category with the frequency information is presented in Table 1.

**Table 1:** Variations of the proverb in COCA.

**Classification**	**Variation types**	**Example**	**Frequency**	**Proportion (%)**
Canonical use	Addition	There are two sides to the economic coin	84	6
	Substitution	There are both sides of that coin	205	15
	Reduction	A coin with two sides	193	14
Non-canonical use	Addition	On the other side of the political coin	250	19
	Substitution	There are two sides to every story	384	29
	Reduction	A two-sided coin	218	17
Total			1,334	100

For TECCL, we set *coin* as the search word and find that 265 instances are relevant to the proverb. Different from the natives, almost all English learners prefer to use the proverb in the form *Every/a/each/the/one coin has two sides*. In other words, for English learners in China, the canonical use of this proverb is *Every coin has two sides*, which accounts for 72% of all the 265 instances. We also searched in TECCL “^*^ has two sides” to see which word can replace *coin* and found that 47 cases of *Everything has two sides*, as shown in Table 2. In other words, Chinese EFL learners are prone to use proverbs in a more mechanical way with little flexibility.

**Table 2:** Variations of the proverb in TECCL.

**Classification**	**Example**	**Frequency**	**Proportion (%)**
Canonical use	Every/a/each/the/one coin has two sides	265	85
Non-canonical use	Everything has two sides	47	15
	Total	312	100

## Results

It seems that proverbs are rarely used in their full original form in a specific context, but rather they are often tailored for communication purposes. The following section presents the variants of *There are two sides to every coin* in COCA and TECCL.

### Canonical use of the proverb in COCA

From COCA, it can be summarized that native speakers usually use addition, substitution, and reduction to alter a proverb, be it canonical or non-canonical.

Addition refers to the insertion of other linguistic units into the original proverb, and the unit is often an adjective, a noun, or an adverb. Nunberg et al. (1994) pointed out that some proverbs need to include more elements to expand their meaning with an aim to fit in each communication context. A common variant form is the addition of adjectival or noun modifiers that further concretize the proverb, as shown in (1)–(4):

(1) Energy and the environment are two sides of the same *policy* coin, and environmental issues are an indispensable part of our national energy conversation (COCA, 2012, WEB).
(2) This imbroglio proves that there are two sides to the *strategic* coin and that today's vital alliance can become tomorrow's crushing burden (COCA, 2000, ACAD).
(3) Every coin *and almost every story* has two sides, and so it is with the story (COCA, 2013, ACAD).
(4) There are *definitely* two sides to the coin, I just shared one to try to get a conversation started (COCA, 2012, BLOG).

The nominal modifier *policy* is added in sentence (1), in which the core word *coin* is influenced by the modifier and its meaning is metaphorized. The feature of a coin is projected onto “policy,” highlighting the dichotomy of environment and energy issues in policy. In sentence (2), the adjective *strategic* is added to extend the feature of a coin to the realm of strategy, emphasizing the positive and negative sides of a strategy. The addition of the noun phrase *almost every story* in the sentence (3) makes the analogy between a coin and a story possible. In sentence (4), the adverb “definitely” is inserted for emphasis.

Substitution refers to the replacement of a word in the original proverb by another without changing the syntactic structure, as shown in (5) and (6):

(5) There are two sides to *that* coin. One is do you keep the player that will help you get there (COCA, 2011, TV/M).
(6) Republicans and Democrats are really just two sides of *the same* coin, and neither of which has our best interests at heart (COCA, 2012, WEB).

In sentence (5), *every* in the original proverb is replaced by *that*. This demonstrative adjective has a referential function, and the reader can determine the reference of “coin” through the context. The replacement of *every* with *the same* in sentence (6) is to shift the focus of the proverb. While *every coin* seems to emphasize the universality of the truth of the proverb, *the same coin* stresses the two sides are identical in their function.

Reduction refers to the removal of certain elements from the original proverb without changing its meaning. The reduced proverb was usually a phrase containing the core words *coin* and *sides*, as shown in (7)–(8) below:

(7) You see, then, that a public enterprise is *a coin with two sides*. On one, the figure of a busy worker (COCA, 2012, WEB).
(8) Douglas Kennedy looks at *both sides of that tax coin* (COCA, 2004, SPOK).

The reduced form in sentence (7) is a phrase, and the use of *coin* as the head of the phrase is intended to highlight the two sides of the business. The reduced variant requires one to engage in cognitive processing for insinuation by extracting the main components of the proverb and creating a proverb activation set that is maximally related to the original proverb. In this way, the proverb can be identified and understood in a variety of contexts. This, of course, requires language users to have a relevant knowledge of proverbs and an awareness of linguistic creativity. It should be noted that the three types of variations mentioned above are not mutually exclusive and that many proverbs in COCA often involve two types of variation patterns. For example, sentence (8) is a shortened version of the original proverb, but with the addition of the thematic cue *tax*.

### Non-canonical use of the proverb in COCA

Non-canonical use is defined here as the proverb variant in which only one core word, i.e., *coin* or *sides*, is preserved. In non-canonical use, there are also three subcategories of alteration, i.e., addition, substitution, and reduction.

According to Langlotz (2006, p. 266), additions can be further divided into two categories: “literal-scene manipulation” and “topic indication.” Literal-scene manipulation is mainly used for effectively encoding the target concept by ways of adapting the literal scene. The manipulation of literal scenes is driven by the specific context, which in turn triggers a change in the meaning of the proverb. Here, the manipulation of literal scenes is mostly done by adding adjectives before the *side*, as shown in (9)–(11):

(9) Boy, that sounds lousy, right? Other side of that coin, the *positive side*, ski areas are loving it. Places like Jay Peach (COCA, 2009, SPOK).
(10) Their style affected us enough to have us on the *wrong side* of the coin, said Otis Thorpe, who got into a shoving scene with New York (COCA, 1993, NEWS).
(11) But the *flip side* of the coin is believers who get down on themselves for not doing what others are doing (COCA, 2012, BLOG).

Carter (2004, p. 132) notes that proverbs can be commented on positively or negatively by adding modifiers to them. Sentence (9) uses *positive* to modify the *coin*, indicating the right side of the coin, a metaphor for the good side of things. As pre-coded cognitive micro-models, proverb prototypes serve as the basis for the concretization of the target concept. In this sentence, the core word *sides* in the original proverb pre-codes two literal scenarios: positive side and negative side, and the added modifier explicitly selects one of the pre-coded scenarios. Sentences (10) and (11), on the other hand, are negative literal scenes. In our data, most of the added adjectives are negative literal scenes, with the most frequent adjectives being *flip* and *opposite*.

Topic indications are adjectives or nouns that denote themes, as shown in (12) and (13):

(12) On the other side of the *political coin*, Richard Nixon most liked to play his presidential golf within the secure confines of (COCA, 1995, ACAD).
(13) The opposite side of the *leadership coin*, shyness or inhibition, has been extensively analyzed by Jerome Kagan in his research (COCA, 1995, ACAD).

The addition of the adjectival modifier *political* in sentence (12) is a thematic cue. Syntagmatically, *political* and *coin* cannot occur together. However, here “political” modifies the metaphorical meaning of the word “coin”, i.e., “a potentially two-faced position.” “Political” refines the category of “position,” or more precisely, a position related to politics. The word “leadership” in the sentence (13) also suggests a theme. The use of this premodifier makes perfect sense in terms of the metaphorical meaning of the “coin.” Because a coin has “two sides,” the positive side of the matter is “leadership” and the negative side is “shyness of personality.” Therefore, the metaphorical meanings of “leadership” and “coin” aim to relate leadership to the metaphorical meaning of coin: “a personality with two sides.”

One common substitution of components in a proverb is the use of synonyms. These synonyms also have the function to trigger the overall meaning of the proverb. There is a wide range of works where synonyms are used to replace proverbial components, such as in this proverb where *coin* is replaced by other words with more contextual meanings, as shown in (14)–(16):

(14) We forget that there are two sides to *every story*, because it tends to complicate things (COCA, 2012, WEB).
(15) Description: There are two sides to *every conflict*. Nothing is ever black-and-white, there are only many shades (COCA, 2012, BLOG).
(16) On the other hand, there are two sides to *every argument*, and the Contract is no exception (COCA, 1995, ACAD).

The users' intention of a proverb may be clearer if the core word of the original proverb is replaced by a word of more specific meanings. These substitutes are also “thematic indicators” (Langlotz, 2006), which reflect conceptual integration. The replacements must share the same conceptual features with *coin*, such as *story, conflict*, and *argument* in sentences (14), (15), and (16). The similarity between the concepts that these words represent is that they are two-sided, and that is consistent with the conceptual features of a coin.

Proverb reduction for non-canonical use is the reduction of the original proverb to a phrase or even a compound containing only one core word, as shown in sentences (17) and (18):

(17) This won't destroy Google. But Google is just one side of the coin and we've had a *one-sided coin* for the last decade and a half (COCA, 2012, BLOG).
(18) To attend schools that are under-budgeted and overpopulated. The current Philippine economy is a *two-sided coin* and I am one of the struggling youth balancing on the rim (COCA, 2016, MAG).

Sentences (17) and (18) exemplify a particular type of variation in English proverbs, namely “constituent reassignment,” which refers to a change in the lexical category of a constituent in a proverb, such as turning a noun (*side*) into an adjective (*sided*). Actually, proverbs often form the basis for phraseological units. For another instance, the phrase *early bird* in *early bird ticket* and *early bird discount* comes from the proverb *early bird gets the worm*. The deletion of *gets the worm* makes the original proverb into a noun phrase, while the meaning remains the same as the original form. The reduced form demonstrates a balance between the economy of language and ease of communication.

### Proverb use in TECCL

For EFL learners, their uses of the proverb present quite a different picture. In TECCL, we find that Chinese EFL learners typically use the proverb in the canonical form *Every/a/each/the/one coin has two sides* as represented in Sentence (19), while the non-canonical usage is represented by Sentences (20) and (21):

(19) But, just as *every coin has two sides*, microblogs might give rise to some problems as well (TECCL).
(20) As *a coin has two aspects*, the Chinese learning style has advantages and disadvantages (TECCL).
(21) In my opinion, *as everything has two sides*, we should treat online shopping more seriously (TECCL).

The EFLs and the natives differ a lot in their use of this proverb. First, the EFLs prefer to use the canonical form including both *coin* and *sides* and the natives prefer the non-canonical form with either *coin* or *sides*. Second, the most common canonical use of this proverb for natives is *there-be* construction, for instance, *There are two sides of that coin*; however, the canonical usage of EFLs is SVO construction, like *Every coin has two sides*. Finally, the natives use the proverb much more flexibly than the EFLs. The native speakers are more flexible in using non-canonical use, accounting for 65% of the total variant forms. Among them, addition, substitution, and reduction each account for 19, 29, and 17%, respectively. Non-canonical use accounts for only 15% of the total variants displayed by EFL learners, indicating a less-than-excellent performance in this area. To better compare the differences in the use of proverb variants between native speakers and EFL learners, we use the prop-test in R language to conduct a chi-square test on the frequency of their use. Our analysis shows that EFL learners significantly overuse canonical use (χ^2^ =124.92, *p* ≤ 0.05) and underuse non-canonical use (χ^2^ =1438.1, *p* < 0.05). Additionally, EFL learners' non-canonical use is relatively uniform, making it impossible to further refine the categories. Overall, our findings indicate that EFL learners do not exhibit as much diversity in their use of proverb variants as native speakers, whether in canonical or non-canonical use.

## Discussion: proverb variation, linguistic creativity, and cognitive creativity

As mentioned above, the EFLs have their own canonical form of this proverb *every/a/one/each coin has two sides*, different from the natives who prefer to use the *there-be* construction. The EFLs' choice of this particular syntactic structure may be due to the syntactic differences between Chinese and English languages. As we know, *there-be* structure is a unique syntactic structure of English and it will usually be transformed to a SVO structure when translated into Chinese because SVO word order is the predominant one in Chinese syntactic structure. That being said, to some extent, the EFLs prefer to translate the meaning of the proverb into English in a word order with typical Chinese characteristics. The main reason is that the native (L1) language plays an important role in L2 learning (Bialystok and Miller, 1999).

It is clear that Chinese EFLs tend to stay within a limited number of variant forms, which indicates that they are relatively inflexible in using the proverb. The main reason may be that Chinese learners only remember the original form and literal meaning of the proverb, but dare not adjust it in a more dynamic and flexible way. From the perspective of construction grammar, once the schematic construction in a learner's mind is fully formed, he or she can concretize the abstract schematic construction (Goldberg, 2019). In other words, there are slots in a schematic construction that can be filled with specific elements, resulting in variations of a proverb and adding more information. In this sense, it seems what Chinese learners have mastered is a concrete construct of the proverb, rather than an abstract schematic construction this proverb represents. Multiple levels of constructions are supposed to be activated in the process of language production (Goldberg, 2019, p. 140), and native speakers are more adept at using proverbs in a wide range of contexts to create innovative expressions, but Chinese EFL learners seem to fail to generalize the schematic form of the original proverb.

This inflexibility reflects that there is room for Chinese EFL learners to improve their sociolinguistic competence when using English proverbs. The ability to change proverbs concerning their use in different contexts shows learners' awareness of registers, which is an important element of sociolinguistic competence (Charteris-Black, 1995). Research shows that native speakers use proverbs as “a significant rhetorical force in various modes of communication” (Mieder, 2004, p. 1), so they can change the proverb in different situations, such as friends' chats, political speeches, and influential mass media. It seems that Chinese EFL learners lack a sense of register when using proverbs. However, this does not mean that EFL learners lack linguistic creativity. Creativity is an essential property of human cognition (Ward et al., 1997). To some extent, EFL learners' linguistic creativity may be influenced by their native language, culture, and L2 language proficiency (Leung and Chiu, 2010; Cook and Bassetti, 2011). It is found that language proficiency is the one of main factors that have a positive impact on creative performance, and more proficient EFL learners show better performance in originality in language use than their less proficient counterparts (Kharkhurin, 2011). Therefore, it may be worthwhile to test that EFL learners of different L2 language proficiency show different linguistic creativity in their use of proverbs, but this cannot be done here due to the limit of TECCL.

Proverb variation reflects the paradox of partial productivity of forms and a balance between linguistic constraint and linguistic creativity. Goldberg (2019, p. 3–4) proposes the paradox of the “partial productivity of linguistic constructions,” which means the productivity of construction was constrained to various degrees. That means it had limited innovativeness. Goldberg argued that language users need to balance expressiveness and validity while adhering to the rules of the linguistic community. There is a link between old and new information that contributes to the formation of a network of sentences. The prototypes and variants of proverbs reflect some of the productive features of constructions. The types of variation in proverbs (e.g., canonical and non-canonical use) and the various ways of variation (e.g., addition, substitution, and reduction) are innovative according to the purpose of communication, but their innovation is also bound by the semantic and formal constraints of the original proverbs. In the case of this proverb, in the prototype use, the original form “two sides to every coin” can trigger new proverbial forms by the methods of adding, substituting, and reducing. The new variations (e.g., two sides of the same policy coin) are to some extent bound by the original proverb (keeping the core vocabulary unchanged). For example, the addition of modifiers makes the semantics more specific, while the form is converted into a phrase and that indicates a certain degree of innovation. In non-canonical usage, the new forms are more formally and semantically innovative and less constrained by the original proverb, but still have identifiable associations, such as “one-sided coin.”

Linguistic creativity used to be seen as the ability of creative writers, such as Shakespeare's new words. However, linguistic innovation is found not only in literary language but also in the everyday use of language. Labov (1973, p. 30) points out that instead of complaining about the variability of word meanings, we should acknowledge that people have an extraordinary ability to describe the world in creative ways. Carter's (2004) book *Language and Creativity* explores the creativity of language in everyday life. Many linguistic games in language reflect language creativity. For instance, *once a pun a time* is a play of the idiom *once upon a time*. This creates a refreshing effect that conveys both the message and the wit, which conveys a kind of linguistic creativity.

It should also be noted that linguistic creativity is one of the linguistic manifestations of the creative potential of human beings. Creativity is the assumption that anyone can create something new out of something old, reflecting the understanding of creativity as the potential ability to create novel, appropriate, and effective outcomes through purposeful behavior in a given context (Kampylis and Valtanen, 2010). Therefore, creativity is considered the creative potential of each individual. In everyday life, this innovative power is manifested in creative acts, and Carter (2004, p. 29) considers two main characteristics of innovative potential, novelty, and appropriateness. Novelty refers to something new and unexpected, usually a departure from the original. Körtvélyessy et al. (2022), while discussing the relationship between creative potential and creativity in word formation, states that creativity in word formation is a manifestation of creative potential in the linguistic domain. Similarly, proverb variation is also a manifestation of creative potential in linguistics, and it also reflects the novelty and appropriateness of the creative potential. Novelty refers to the proverb's semantic and formal departure from the proverbial prototype, and appropriateness refers to the proverb variant's conformity to the communicative purpose and contextual needs of the subject.

Proverb variation is an innovative linguistic expression of creative potential, indicating cognitive creativity. Cognitive innovation is the source of linguistic creativity (Leikin et al., 2014; Vaid et al., 2015, p. 53–86). Langlotz (2016) considers innovative cognitive thinking as the ability of people to create new and unconventional knowledge from a familiar, established stock of knowledge. Similarly, Guilford (1967) defines creative thinking as a person's ability to generate new ideas or products, which consists of divergent and convergent thinking. Divergent thinking is defined as the process that allows people to generate as many responses as possible to a particular trigger or problem (Mumford et al., 1991). In examining the proverb variants in the COCA corpus, we find that native speakers can generate many variants for a proverb in a particular context. Therefore, proverb variants were hardly spontaneous, but rather emerge from creative thinking. From the perspective of cognitive psychology, proverb variation, and comprehension involve complex cognitive processes, including such cognitive tasks as activating proverb prototypes in the brain and integrating them with other predetermined symbolic units and construal schemas, which Richard (1997, p. 88) calls the proverbial task. When people realize that the literal meaning of the word ‘coin' does not fit the context, they will combine various resources and relevant contextual information to shape a new meaning, thus producing a metaphorical meaning. The metaphorical meaning not only maps the contextual meaning but also expands the derivational meaning of the coin. From the speaker's perspective, the production of variants also involves creative cognitive processes. The speaker first searches for a suitable proverb in the knowledge base, gets Familiar with the context, and generates an affective attitude (e.g., the opposite side). Finally, the original proverb is matched with the context to produce a reasonable variant of the proverb, such as “the opposite side of the leadership coin.” Kirshenblatt-Gimblett (1973) argues that we should not focus on the “base meaning” of the proverb, but rather consider the larger picture provided by the proverbial performance, including the speaker's evaluation of the situation and the intention of using the proverb.

## Conclusion

Proverbs are usually regarded as structurally fixed expressions, but they may manifest many variations in language usage. The present study finds that proverb variants can be divided into two types: canonical and non-canonical uses according to whether the core words are retained or not. Under each of the two types, there are three specific methods of variation, namely addition, substitution, and reduction. The variation of proverbs is closely related to linguistic creativity. From the perspective of linguistic creativity, proverb variation is the creative manipulation of proverbs to fit the context, which is a form of linguistic creativity that reflects the cognitive creativity of individuals. Chinese EFL learners tend to use the proverb in a more mechanical way, which shows their inflexible use of proverbs and inferior sociolinguistic competence and metaphoric competence. Consequently, we need to introduce the concept of linguistic creativity in our classroom teaching.

## Data availability statement

The original contributions presented in the study are included in the article/Supplementary material, further inquiries can be directed to the corresponding author.

## Author contributions

JW: writing the draft of the manuscript. WZ: data collection and methodology. BS: designed the research, revised the writing, and improved the manuscript. All the authors contributed to the article and approved the submitted version.
